# Immunohistochemical Analysis of Paraoxonases and Chemokines in Arteries of Patients with Peripheral Artery Disease

**DOI:** 10.3390/ijms160511323

**Published:** 2015-05-18

**Authors:** Anna Hernández-Aguilera, Julio Sepúlveda, Esther Rodríguez-Gallego, Maria Guirro, Anabel García-Heredia, Noemí Cabré, Fedra Luciano-Mateo, Isabel Fort-Gallifa, Vicente Martín-Paredero, Jorge Joven, Jordi Camps

**Affiliations:** 1 Biomedical Research Unit, Hospital Universitari de Sant Joan, Institut d’Investigacio Sanitaria Pere Virgili, Universitat Rovira i Virgili, Reus, Catalonia 43201, Spain; E-Mails: anna.hernandeza@gmail.com (A.H.-A.); esther.rodriguez@grupsagessa.com (E.R.-G.); maria.guirro@gmail.com (M.G.); ghanabel@gmail.com (A.G.-H.); noemi.cabre@gmail.com (N.C.); fedra.luciano@gmail.com (F.L.-M.); ifort@lrsud.cat (I.F.-G.); 2 Service of Angiology, Vascular Surgery and Endosurgery, Hospital Universitari Joan XXIII, Institut d’Investigacio Sanitaria Pere Virgili, Universitat Rovira i Virgili, Tarragona, Catalonia 43005, Spain; E-Mails: juliosepulvedag@ymail.com (J.S.); vparedero.hj23.ics@gencat.cat (V.M.-P.)

**Keywords:** CCL2, chemokine receptors, chemokines, immunohistochemistry, paraoxonases

## Abstract

Oxidative damage to lipids and lipoproteins is implicated in the development of atherosclerotic vascular diseases, including peripheral artery disease (PAD). The paraoxonases (PON) are a group of antioxidant enzymes, termed PON1, PON2, and PON3 that protect lipoproteins and cells from peroxidation and, as such, may be involved in protection against the atherosclerosis process. PON1 inhibits the production of chemokine (C–C motif) ligand 2 (CCL2) in endothelial cells incubated with oxidized lipoproteins. PON1 and CCL2 are ubiquitously distributed in tissues, and this suggests a joint localization and combined systemic effect. The aim of the present study has been to analyze the quantitative immunohistochemical localization of PON1, PON3, CCL2 and CCL2 receptors in a series of patients with severe PAD. Portions of femoral and/or popliteal arteries from 66 patients with PAD were obtained during surgical procedures for infra-inguinal limb revascularization. We used eight normal arteries from donors as controls. PON1 and PON3, CCL2 and the chemokine-binding protein 2, and Duffy antigen/chemokine receptor, were increased in PAD patients. There were no significant changes in C–C chemokine receptor type 2. Our findings suggest that paraoxonases and chemokines play an important role in the development and progression of atherosclerosis in peripheral artery disease.

## 1. Introduction

Lower-extremity peripheral artery disease (PAD) is an important health problem that is associated with severe impairment of different arterial territories. Indeed, PAD is a predictor of substantial coronary and cerebral vascular risk [1,2]. The disease prevalence increases with age and, in people over the age of 55 years, it is estimated to be about 20% [3,4,5,6]. Atherosclerosis affects wide portions of numerous arteries in the lower extremities of PAD patients. This is the effect of a sustained and silent progression of the disease in which appropriate and effective prevention measures are applied too late, or not implemented at all [3,4,5,6,7,8].

Oxidative damage to lipids and lipoproteins is implicated in the development of atherosclerotic vascular diseases, including PAD [9,10]. The paraoxonases (PON) are a group of antioxidant enzymes that protect lipoproteins and cells from peroxidation and are involved in the atherosclerosis process and, consequently, in vascular diseases [11]. The PON family contains three enzymes: PON1, PON2 and PON3, the genes of which are located adjacent to each other on chromosome 7q21–22 [12,13]. PON1 and PON3 are found in many tissues, as well as in blood, where they are associated with high-density lipoproteins (HDL). Conversely, PON2 is exclusively intracellular [14,15,16,17]. Pioneer studies reported that oxidized low-density lipoprotein uptake by macrophages in tissue culture and *in vivo* increases the production of the inflammatory chemokine (C–C motif) ligand 2 (CCL2). The consequence is the stimulation of arterial fatty streak formation, which is the progenitor of atheroma. PON1 has been shown to inhibit these alterations [18,19,20]. Chemokines, CCL2 in particular, are central to the vascular inflammatory response in mediating monocyte recruitment into the arterial wall [21,22]. We have previously reported that PON1 and CCL2 are ubiquitously distributed in mouse tissues, suggesting a joint localization and combined systemic effects [23]. Clinical data suggest that circulating CCL2 concentrations or serum PON1 activity are important biomarkers of a variety of diseases involving inflammatory response to an increased oxidative stress [24,25,26,27,28,29].

Previous studies from our group found that serum PON1 activity and concentration were significantly lower, and CCL2 concentration higher, in PAD patients compared to controls, while the combination of plasma CCL2 and PON1-related variables, discriminated controls from patient almost completely [30]. In addition, we observed an increase in serum PON3 concentration in PAD patients, relative to the healthy population [31]. However, data on the protein expression of these molecules at the lesion level in patients with PAD are scarce. The aim of the present study was to quantify the immunohistochemical localization of PON1, PON3, CCL2 and CCL2 receptors in a wide series of patients with severe PAD.

## 2. Results

Patients with PAD did not significantly differ from the control group in age and gender distribution. The patient group had a significantly higher percentage of smokers, and lower serum cholesterol and low-density lipoprotein (LDL) cholesterol concentrations. We did not observe significant difference in any of the other standard biochemical and hematological variables. The circulating levels of CCL2 and 8-isoprostanes (a marker of oxidative stress) were significantly increased in PAD, while serum PON1 concentrations and activities were decreased (Table 1). C-reactive protein (CRP) protein levels were not significantly increased in our patients, a finding probably related to that they were treated with salicylates and antiplatelet agents.

**Table 1:** Selected descriptive characteristics and laboratory variables in participants.

Parameter	Control (*n* = 8)	PAD (*n* = 66)	*p*-Value
Clinical characteristics			
Age, years	66 (30–76)	70 (62–77)	0.223
Male, *n* (%)	5 (62.5)	55 (85.9)	0.094
Smokers, *n* (%)	1 (14.3)	16 (31.4)	**0.048**
Complete blood count			
Red blood cells, ×10^12^/L	4.32 (3.18–4.47)	3.67 (3.14–4.24)	0.449
Hemoglobin, g/dL	12.46 (9.99–13.28)	10.85 (9.45–12.93)	0.468
Leukocytes, ×10^9^/L	9.22 (8.58–10.17)	9.89 (7.44–12.20)	0.668
Platelets, ×10^9^/L	227.5 (163.7–246.2)	312.5 (199.0–419.0)	0.080
Biochemical variables in serum or plasma			
Glucose, mmol/L	5.77 (5.11–6.77)	6.38 (5.11–8.83)	0.406
Total cholesterol, mmol/L	4.77 (3.87–6.39)	3.39 (2.90–4.47)	**0.030**
HDL cholesterol, mmol/L	1.24 (0.98–1.40)	0.93 (0.83–1.20)	0.074
LDL cholesterol, mmol/L	3.54 (3.11–4.42)	1.95 (1.68–2.69)	**0.001**
Triglycerides, mmol/L	1.47 (1.13–2.15)	1.31 (1.00–1.87)	0.449
Fibrinogen, g/L	5.51 (4.48–7.54)	6.96 (5.34–8.11)	0.237
C-reactive protein, mg/L	6.1 (0.6–7.2)	8.1 (2.7–16.0)	0.147
Total proteins, g/L	65 (55–68)	60 (55–69)	0.743
CCL2, ng/L	373.4 (255.2–431.8)	622.8 (472.7–898.4)	<**0.001**
PON1, mg/L	75.4 (56.7–143.8)	25.2 (18.4–35.8)	<**0.001**
PON3, mg/L	1.95 (1.51–2.50)	1.73 (1.43–2.27)	0.490
8-Isoprostanes, ng/L	14.2 (2.0–37.2)	100.8 (37.6–314.7)	<**0.001**
PON1 lactonase activity, U/L	5.69 (5.02–6.29)	3.04 (2.11–3.73)	<**0.001**

The bold numbers highlight the statistically significant differences.

The histological and immunohistochemical analyses of the peripheral arteries revealed that PAD patients had a significantly thicker tunica intima relative to the tunica media of the artery wall (termed the intima-media, or I/M ratio). There were significant increases in the percentage positive staining for PON1, PON3, CD68 antigen (a marker of macrophages), CCL2, and also in the CCL2 receptors termed chemokine-binding protein 2 (CCBP2, also termed D6), and Duffy antigen/chemokine receptor (DARC). We did not observe any significant change in C–C chemokine receptor type 2 (CCR2) staining relative to controls (Table 2). Similar results were obtained when smokers were excluded from the PAD group (Table S1).

**Table 2:** Differences in selected variables between control individuals and PAD patients.

Parameter	Control (*n* = 8)	PAD (*n* = 66)	*p*-Value
IMT (mm)	1.00 (0.70–1.30)	1.29 (1.00–1.74)	0.150
I/M ratio	0.16 (0.13–0.65)	2.10 (1.33–3.22)	<**0.001**
% PON1 staining	1.70 (1.54–3.72)	11.19 (7.25–20.81)	<**0.001**
% PON3 staining	0.55 (0.22–0.73)	3.25 (2.01–4.37)	<**0.001**
% CCL2 staining	2.26 (0.36–3.65)	30.75 (9.63–44.41)	<**0.001**
% CCR2 staining	18.29 (7.02–27.56)	22.99 (13.21–42.71)	0.263
% CD68 staining	1.10 (0.65–2.88)	4.57 (2.40–9.24)	**0.007**
% D6 staining	0.83 (0.22–12.9)	41.21 (24.55–58.39)	<**0.001**
% DARC staining	3.29 (2.01–5.06)	37.26 (18.06–51.85)	<**0.001**

IMT: Intima-Media thickness. Results are shown as medians and interquartile ranges. Staining for chemokine (C–C motif) ligand 2 (CCL2), C–C chemokine receptor type 2 (CCR2), cluster of differentiation 68 (CD68), Duffy antigen/chemokine receptor (DARC), chemokine-binding protein 2 (D6), paraoxonase-1 (PON1) and paraoxonase-3 (PON3) were measured as the area of positive staining and expressed as percentage of the total area examined using the image analysis system (see text for details). The bold numbers highlight the statistically significant differences.

**Figure 1:**
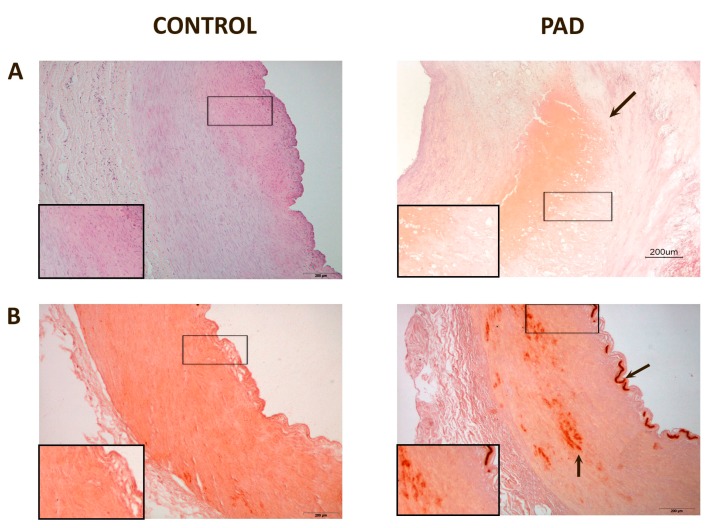

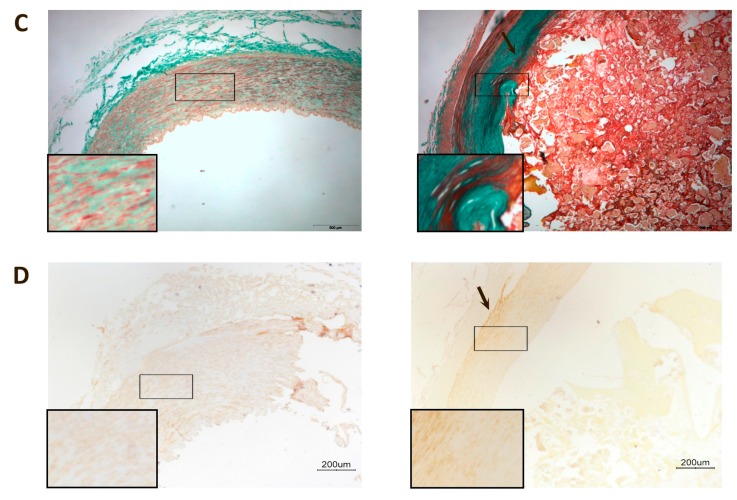
Representative histological images of peripheral arteries: (**A**) Arteries stained with Hematoxylin-Eosin. The intima in affected arteries was thicker and replete with cholesterol deposits and inflammatory cells (arrow). Magnification 20×; (**B**) Alizarin Red staining to detect the presence of calcium. There were calcium deposits in affected arteries located, mainly, in the media and, in some cases, calcium was observed in the internal elastic lamina (arrows). Magnification 20×; (**C**) Masson’s Trichrome stain showing, in affected arteries, an infiltration of smooth muscle cells from the media to the intima (arrow). The lumen shows partial obstruction. Magnification 40×; (**D**) Actin staining to detect the presence of smooth muscle cells. The arrow shows the area of infiltration of these cells from the media to the intima. Magnification 20×. The inserts show higher magnification (100×) images of the indicated areas.

Affected arteries had severe alterations compared to the normal artery histology (Figure 1). The intima was thicker and had extensive deposits of cholesterol and inflammatory cells. Calcium deposits were clearly identified in the media. Masson’s trichrome stain was used to evaluate the arteries’ architecture which, in affected arteries, highlighted an infiltration of smooth muscle cells from the media into the intima, or perhaps a loss of muscle cells from the media and increase in connective tissue, and greater obstruction of the arterial lumen.

**Figure 2:**
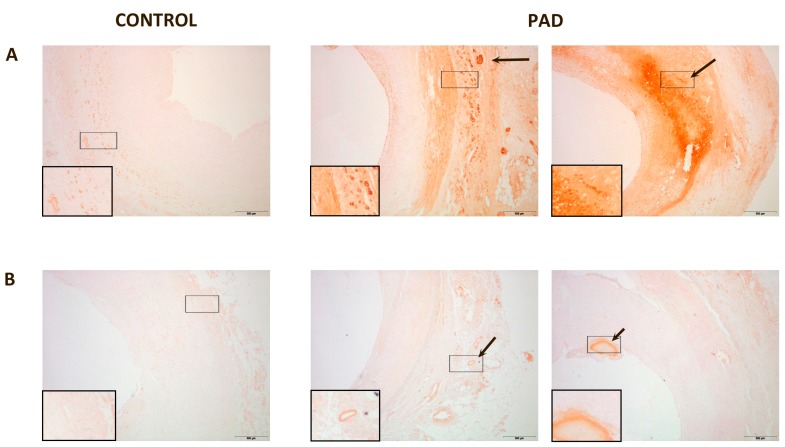
Representative immunohistochemical images for paraoxonase-1 (PON1) and paraoxonase-3 (PON3) staining of peripheral arteries: (**A**) PON1 expression in normal artery was almost undetectable, and located in the media and adventitia. PON1 had two types of localization in affected arteries: when the intima was not very thick, PON1 was located in the adventitia and media of the vessels (arrow). When the intima was disorganized and with cholesterol deposits, PON1 was expressed in the lesion site (arrow); (**B**) PON3 expression was undetectable in normal tissue whereas, in affected arteries, PON3 was located in the adventitia or in the injury sites of the intima (arrow). Magnification 20×. The inserts show higher magnification (100×) images of the indicated areas.

In normal arteries, PON1 expression was low and located in the intima and in the adventitia. PON3 expression was imperceptible. Conversely, in the arteries of PAD patients, PON1 and PON3 expression were higher. PON1 presented two types of localization: (1) when the intima was only moderately enlarged, PON1 was located in the adventitia vessels and the media; (2) when the intima was disorganized and with cholesterol deposits, PON1 was found surrounding the cholesterol crystals at the site of the lesion. In affected arteries, PON3 was found in the adventitia or in the injury sites of the intima (Figure 2). Areas of CD68 staining had a similar spatial distribution than those of paraoxonases and CCL2 (Figures S1 and S2).

**Figure 3:**
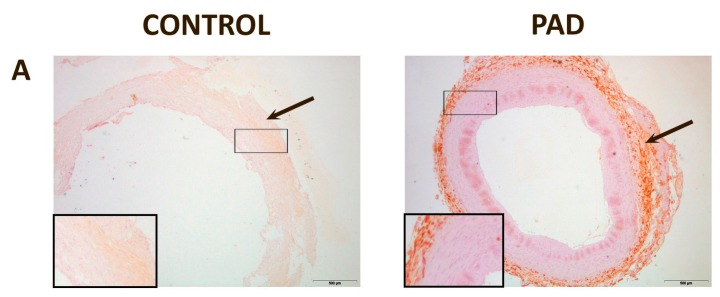

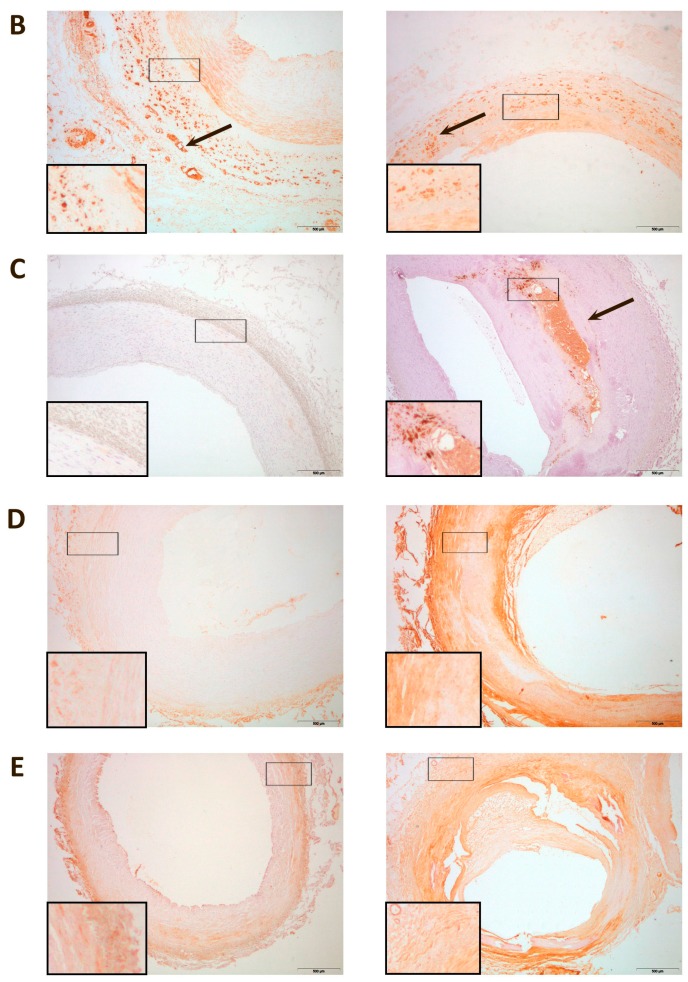
Representative immunohistochemical images for inflammatory markers in peripheral arteries: (**A**) Chemokine (C–C motif) ligand 2 (CCL2) was expressed in the adventitia in normal and affected arteries (arrow); (**B**) C–C chemokine receptor type 2 (CCR2) was expressed, mainly, in the media in normal and affected arteries. However, it can also be found in the intima and in adventitia of the vessels (arrow); (**C**) Cluster of differentiation 68 (CD68) was mildly expressed in control arteries while, in affected arteries, the expression was higher and located, mainly, in the intima (arrow); (**D**) Chemokine-binding protein 2 (D6) expression was found, mainly, in the adventitia; (**E**) Duffy antigen/chemokine receptor (DARC) was found, mainly, in the media, although it was observed as well in the adventitia and/or intima of some vessels. Magnification 20×. The inserts show higher magnification (100×) images of the indicated areas.

In normal arteries, CCL2 was mildly expressed in the adventitia, while CCR2 was found mostly in the media, with weaker expressions in the adventitia and intima. CD68, D6 and DARC expressions were mild. Conversely, the arteries of PAD patients had higher expressions of CCL2, CD68, D6 and DARC. CCL2 was found mostly in the adventitia while CCR2 was found mostly in the media, with weaker expressions in the adventitia and intima, as found in normal arteries. CD68 expression was observed mostly in the thickest areas of the intima. DARC was located mostly in the media, although it could also be found in the adventitia and/or intima of the vessels. D6 was found mostly in the adventitia (Figure 3).

## 3. Discussion

The present study shows (by immunostaining) that paraoxonases, CCL2 and several CCL2 receptors are increased in peripheral arteries with indications of atherosclerosis. This could be a response to increased cellular oxidative stress as well as the migration of monocytes. In PAD patients, we observed an increased CD68 staining which is a specific marker of macrophages. Macrophage mitochondrial oxidative stress plays a major role in atherosclerosis via mechanisms involving the NF-κB-CCL2 pathway [32]. Paraoxonases prevent oxidative stress by reducing the amount of oxidized LDL in the circulation as well as the vessel wall. This, in turn, reduces monocyte infiltration into the vessel wall and, as such, is anti-inflammatory [33,34]. The protein expression of this enzyme has been observed in many tissues in humans [35] and mice [23]. PON1 reduces macrophage oxidation of LDL as well as macrophage oxidative stress, and increases cholesterol efflux from macrophages to high-density lipoprotein (HDL), thus reducing foam cell formation and, as a consequence, the development or progression of atherosclerosis. Therefore, the increase in PON1 staining found in this study could indicate that a protective response to increased oxidative stress was occurring in the macrophages of the diseased arteries. For example, it is of considerable note that PON1 expression was found surrounding cholesterol deposits in severely diseased arteries, and which strongly supports the hypothesis of a protective role for this enzyme, *i.e.*, that PON1 infiltrates the arterial tissue to combat the deposition of the atherosclerosis-promoting cholesterol. The physiological role of PON3 is still unclear. Results from the present study support previous findings from our group showing increased serum PON3 concentrations in patients with PAD [31]. Studies on cellular expression of this enzyme and the elucidation of its athero-protective role are scarce and inconclusive. PON3 has lactonase but not paraoxonase activity [36,37]. Previous studies reported that PON3 attenuates the oxidation of LDL *in vitro* [38] and that the overexpression of human PON3 decreased atherosclerosis and adiposity in mice [39]. Although the increase in PON3 protein expression in the arteries of PAD patients is quantitatively small, it needs to be taken into account that PON3 is about 100 times more potent per mg of protein than PON1, in protecting LDL against lipid peroxidation [36]. Hence, the increase in the enzyme’s expression in these patients could be of clinical relevance.

In the peripheral circulation, decreased PON1 activities are associated with increased concentration of CCL2 [30], and *in vitro* studies found that PON1 inhibits the production of CCL2 induced by oxidative stress in endothelial cells [20]. However, this inverse relationship is not confirmed at tissue level. Indeed, both molecules are ubiquitously expressed in most tissues and are located in close proximity to one another, suggesting some manner of coordinated function [23,40]. Results of the present study, and previous others, show that the expression of both proteins is increased in the arteries of patients with atherosclerosis [40]. This observation would suggest that the variations in PON1 and CCL2 concentrations in plasma do not necessarily correlate with their roles at the cellular level. Perhaps PON1 protein expression is increased in diseased arteries to counteract oxidative stress and CCL2-induced inflammation. However, this hypothesis has to be confirmed by further studies.

CCL2 is likely to have considerable impact on PAD since the biological function of this chemokine is to induce monocyte migration and, as well, because the arteries with moderate atherosclerosis appear to accumulate CCL2 in response to a variety of pro-inflammatory stimuli [24,30,41,42,43,44]. Atherosclerosis is an inflammatory disease, and the consensus is that CCL2 is involved in its pathogenesis [45]. In the present study, we found increased CCL2 expression in the arteries of PAD patients, together with an increased expression of two of the CCL2 receptors *i.e.*, D6 and DARC. D6 and DARC belong to the poorly-understood chemokine receptors collectively known as atypical or silent. These are G-protein coupled receptors that do not activate conventional signaling events. Conversely, they may internalize, degrade or transport ligands (*i.e.*, they have the potential to create clinically relevant chemokine patterns in tissues) [46]. Their levels of expression have not been explored previously in diseased arteries of PAD patients. The availability of CCL2 may be complicated by potential effects induced by differential expression of the specific receptor CCR2 and the presence of these atypical chemokine receptors. We observed that the expression of these receptors was increased in diseased arteries, and that their histological distributions are not uniform. A pathogenic role is likely, and data suggests that atypical chemokine receptors modify chemokine availability in PAD. Although these receptors have no involvement in cell migration, their modulatory effect on inflammatory response is likely.

Previous studies from our group also reported increased PON1 and PON3 expressions in aortas from patients undergoing coronary or aortic artery bypass grafting [40]. This is relevant, because it suggests that, despite the atherosclerosis burden being higher in PAD, changes inside the tissue are similar at a molecular level. The mechanisms underlying the increased PON1 and PON3 immunohistochemical staining in the arteries of PAD patients cannot be ascertained from the present investigation, but these patients had oxidative stress, as indicated by the elevated serum 8-isoprostanes concentration. Oxidative stress stimulates PPARγ and NF-κB-related pathways [47], and these molecules have been reported to stimulate the expression of paraoxonases [48,49]. However, this increase is in an apparent contradiction with the decrease in the serum levels of the enzymes, and a possible explanation could be an increase in PPARδ expression and decreased PON1 proteolysis. This is the case in a rat model of liver fibrosis that our group published a few years ago [50]. Rats with carbon tetrachloride-induced liver fibrosis had oxidative stress and increased PPARδ gene expression. These alterations were associated to an inhibition of the HDL synthesis and, consequently, a decreased PON1 secretion to the extracellular medium. In addition, the hepatic levels of the protease cathepsin B were decreased, leading to an inhibition of protein degradation. Thus, hepatic PON1 levels were elevated as a consequence of the combination of a decreased HDL secretion, and to an inhibition of lysosomal protein degradation. To ascertain if the same phenomena occur in the arteries of PAD patients requires further studies, but the strong decrease in HDL-cholesterol concentrations observed in our patients is in agreement with this hypothesis.

A caveat of the present study is that we could not analyze PON2 in the arteries of PAD patients. This enzyme plays an important role in the intracellular protection against oxidative stress [14], and new investigations focused in PON2 and PAD should be further pursued.

## 4. Experimental Section

### 4.1. Study Population

Patients with clinically diagnosed PAD were recruited from the outpatient clinics of Hospital Universitari Joan XXIII. Diagnosis was with standard clinical assessments including measurement of the ankle-brachial index (ABI), non-invasive imaging, and angiography when indicated. Symptoms of chronic ischemia were detected using the Fontaine classification, the standardized physician-administered questionnaire that seeks to identify the presence of calf discomfort on exertion, such as walking uphill or walking rapidly [51]. Exclusion criteria from our study were the presence of acute ischemia, signs of infection, renal failure, liver disease, cancer, or autoimmune disease. Portions of femoral and/or popliteal arteries from patients were obtained during surgical procedures for infra-inguinal limb revascularization (*n* = 66). All patients were at Stages III and IV of the Fontaine classification. Eight normal arteries obtained from accident victims and stored at the Blood and Tissue Bank of Catalonia (Banc de Sang i Teixits, www.bancsang.net/es/donants/donacio_teixits.html, Barcelona, Spain) were used as controls. All tissues (patients and controls) were kept at −80 °C until thawed for processing. After thawing, the tissues were rinsed in phosphate buffer to remove residual blood and placed in at least 10 volumes of buffered formalin using a standard protocol for embedding tissue in paraffin wax for subsequent histology slide preparation. Three sections per slide were used for histological and immunohistochemical analyses. A peripheral blood sample was also obtained from each patient (and control individual) at the time of the surgery for biochemical and hematological measurements. The hospital’s Ethics Committee (Institutional Review Board) approved the procedures of the study protocol on 31 July 2014, and written informed consent was obtained from the participants prior to entry into the study (OBESPAD 14-07-31/7proj3).

### 4.2. Biochemical Analyses

Serum concentrations of glucose, cholesterol, HDL cholesterol, triglycerides, fibrinogen, C-reactive protein, total proteins, and complete blood cell counts were performed by standard methods in the Hospital Universitari Joan XXIII. LDL cholesterol concentrations were estimated using the Friedewald formula. Serum concentrations of PON1 and PON3, and EDTA-plasma concentrations of CCL2 were determined by ELISA as previously reported [30,31]. Serum concentrations of 8-isoprostanes were analyzed by Enzyme Immunoassay (Cayman Chemical Co., Ann Arbor, MI, USA). Serum PON1 lactonase activity was analyzed by measuring the hydrolysis of 5-thiobutyl butyrolactone [27]. Inter-assay coefficients of variation were as follows: Glucose, 1.8%; cholesterol, 1.5%; HDL cholesterol, 2.0%; triglycerides, 2.2%; fibrinogen, 7.5%; C-reactive protein, 4.8%; total proteins, 1.3%; LDL cholesterol, 3.5%; PON1, 10.5%; PON3, 12.2%; CCL2, 7.3%; 8-isoprostanes, 10.2%; lactonase, 11.5% (*n* = 20 for each variable).

### 4.3. Histological and Immunohistochemical Analyses

Sections, of 4-µm thickness, were stained with hematoxylin-eosin for arterial histology. Masson’s trichrome stain (Masson’s Trichrome Goldner with light green, Bio Optica, Milano, Italy) was used to assess the structure and extent of fibrosis. Alizarin Red staining (Sigma-Aldrich, Steinheim, Germany) was used to identify the sites of micro-crystalline, or non-crystalline, calcium phosphate salts. The intima and media thicknesses were measured in all histological sections as an estimate of the extent of atherosclerosis. Antibodies against PON1 and PON3 were raised in rabbits using peptides derived from specific sequences of mature PONs, as previously reported [52,53,54]. PON1 and PON3 antibodies were used at a dilution of 1/50 and 1/300, respectively. A previous study already demonstrated that these antibodies were highly specific for PON1 and PON3 [54]. Commercial primary antibodies were purchased: CCL2 (dilution 1/200), CCR2 (dilution 1/100), and D6 (dilution 1/500) from Abcam plc (Cambridge, UK); antibodies against DARC (dilution 1/200) from Abnova (Taipei, Taiwan); and antibodies against CD68 from Dako (Glostrup, Denmark). The appropriate biotinylated secondary antibodies (anti-rabbit, anti-mouse or anti-goat; purchased from Vector Laboratories Inc., Burlingame, CA, USA) were used at a dilution of 1:200. Detection was performed with the ABC peroxidase system (Vector Laboratories, Burlingame, CA, USA) and 3,3'-diaminobenzidine (DAB) peroxidase substrate (Dako). The times of the detection reactions were 4 min for PON1 and PON3, 1 min for DARC, 1.5 min for CCR2 and D6, 10 min for CCL2, and 5 min for CD68. All immunohistochemical sections were counterstained with Mayer’s hematoxylin. Negative control samples were processed identically to the test samples except that the primary antibodies were omitted from the incubation. Representative immunohistochemical images of negative controls in control arteries and arteries from patients with PAD are shown in Figures S3 and S4. The positively-stained area was quantified automatically for each antibody using an image analysis system (AnalySIS^®^, Soft Image System GmbH, Olympus Corp., Munster, Germany), and expressed as percentage of the total area. Initially the colors of the images that have been stained to the molecule of interest were defined. Once these colors were defined, they were automatically detected in all samples. The software analyzed the stained area in relation to the total image area, which is termed phase analysis. The rationale for this method is described in more detail in the Supplementary Methods, and is also available on the Internet [55]. This is a semi-quantitative analysis that measures areas and not intensities. This method is commonly accepted and has been employed previously in several immunohistochemical studies by our group and other authors [23,28,40,54,56,57,58,59]. Inter-assay coefficients of variation were as follows: PON1, 9.6%; PON3, 7.3%; CCL2, 4.5%; CCR2, 5.3%; D6, 6.4%; DARC, 7.1% (*n* = 20 for each variable).

### 4.4. Statistical Analyses

Significance of difference between groups was assessed by the Mann–Whitney *U*-test. Results are expressed as medians and IQR (Interquartile Range). All statistical analyses were performed with the Statistical Package for the Social Sciences, version 22.0 (SPSS Inc., IBM Corp., Chicago, IL, USA).

## 5. Conclusions

In conclusion, PON1 and PON3, CCL2 together with the D6 and DARC receptors are increased in the arteries of patients with PAD. The findings suggest that these molecules may be involved in the development and progression of atherosclerosis in peripheral artery disease.

## Supplementary Materials

Supplementary materials can be found at http://www.mdpi.com/1422-0067/16/05/11323/s1.

## References

[1] Golomb B.A., Dang T.T., Criqui M.H. (2006). Peripheral arterial disease: Morbidity and mortality implications. Circulation.

[2] Newman A.B., Shemanski L., Manolio T.A., Cushman M., Mittelmark M., Polak J.F., Powe N.R., Siscovick D. (1999). Ankle-arm index as a predictor of cardiovascular disease and mortality in the cardiovascular health study. Arterioscler. Thromb. Vasc. Biol..

[3] Criqui M.H. (2001). Peripheral arterial disease—Epidemiological aspects. Vasc. Med..

[4] Mehlsen J., Wiinberg N., Joergensen B.S., Schultz-Larsen P. (2010). High prevalence of peripheral arterial disease in patients with previous cerebrovascular or coronary event. Blood Press..

[5] Gornik H.L., Creager M.A. (2006). Contemporary management of peripheral arterial disease: Cardiovascular risk-factor modification. Clevel. Clin. J. Med..

[6] Hirsch A.T., Criqui M.H., Treat-Jacobson D., Regensteiner J.G., Creager M.A., Olin J.W., Krook H., Hunninghake D.B., Comerota A.J., Walsh M.E. (2001). Peripheral arterial disease detection, awareness, and treatment in primary care. JAMA.

[7] McDermott M.M., Liu K., Greenland P., Guralnik J.M., Criqui M.H., Chan C., Pearce W.H., Schneider J.R., Ferrucci L., Celic L. (2004). Functional decline in peripheral arterial disease: Associations with the ankle brachial index and leg symptoms. JAMA.

[8] Criqui M.H., Langer R.D., Fronek A., Feigelson H.S., Klauber M.R., McCann T.J., Browner D. (1992). Mortality over a period of 10 years in patients with peripheral arterial disease. N. Engl. J. Med..

[9] Strzyżewski K.W., Pioruńska-Stolzmann M., Majewski W., Kasprzak M., Strzyżewski W. (2013). Effect of surgical treatment on lipid peroxidation parameters and antioxidant status in the serum of patients with peripheral arterial disease. Dis. Markers.

[10] Arslan C., Altan H., Beşirli K., Aydemir B., Kiziler A.R., Denli S. (2010). The role of oxidative stress and antioxidant defenses in Buerger disease and atherosclerotic peripheral arterial occlusive disease. Ann. Vasc. Surg..

[11] Abelló D., Sancho E., Camps J., Joven J. (2014). Exploring the role of paraoxonases in the pathogenesis of coronary artery disease: A systematic review. Int. J. Mol. Sci..

[12] Primo-Parmo S.L., Sorenson R.C., Teiber J., la Du B.N. (1996). The human serum paraoxonase/arylesterase gene (*PON1*) is one member of a multigene family. Genomics.

[13] Sorenson R.C., Primo-Parmo S.L., Camper S.A., la Du B.N. (1995). The genetic mapping and gene structure of mouse paraoxonase/arylesterase. Genomics.

[14] Camps J., Marsillach J., Joven J. (2009). The paraoxonases: Role in human diseases and methodological difficulties in measurement. Crit. Rev. Clin. Lab. Sci..

[15] Hine D., Mackness B., Mackness M. (2012). Coincubation of PON1, APO A1, and LCAT increases the time HDL is able to prevent LDL oxidation. IUBMB Life.

[16] Mackness M.I., Durrington P.N., Mackness B. (2004). The role of paraoxonase 1 activity in cardiovascular disease: Potential for therapeutic intervention. Am. J. Cardiovasc. Drugs.

[17] Mackness M.I., Mackness B., Durrington P.N. (2002). Paraoxonase and coronary artery disease. Atheroscler. Suppl..

[18] Mackness M.I., Arrol S., Durrington P.N. (1991). Paraoxonase prevents accumulation of lipoperoxides in low-density lipoprotein. FEBS Lett..

[19] Mackness M.I., Arrol S., Abbott C., Durrington P.N. (1993). Protection of low-density lipoprotein against oxidative modification by high-density lipoprotein associated paraoxonase. Atherosclerosis.

[20] Mackness B., Hine D., Liu Y., Mastorikou M., Mackness M. (2004). Paraoxonase-1 inhibits oxidised LDL-induced MCP-1 production by endothelial cells. Biochem. Biophys. Res. Commun..

[21] Feldmann M. (2008). Many cytokines are very useful therapeutic targets in disease. J. Clin. Investig..

[22] Charo I.F., Taubman M.B. (2004). Chemokines in the pathogenesis of vascular disease. Circ. Res..

[23] Rodríguez-Sanabria F., Rull A., Beltrán-Debón R., Aragonès G., Camps J., Mackness B., Mackness M., Joven J. (2010). Tissue distribution and expression of paraoxonases and chemokines in mouse: The ubiquitous and joint localisation suggest a systemic and coordinated role. J. Mol. Histol..

[24] Rull A., Camps J., Alonso-Villaverde C., Joven J. (2010). Insulin resistance, inflammation, and obesity: Role of monocyte chemoattractant protein-1 (or CCL2) in the regulation of metabolism. Mediat. Inflamm..

[25] Petrkova J., Szotkowska J., Hermanova Z., Lukl J., Petrek M. (2004). Monocyte chemoattractant protein-1 in patients with peripheral arterial disease. Mediat. Inflamm..

[26] Van Wijk D.F., van Leuven S.I., Sandhu M.S., Tanck M.W., Hutten B.A., Wareham N.J., Kastelein J.J., Stroes E.S., Khaw K.T., Boekholdt S.M. (2010). Chemokine ligand 2 genetic variants, serum monocyte chemoattractant protein-1 levels, and the risk of coronary artery disease. Arterioscler. Thromb. Vasc. Biol..

[27] Marsillach J., Aragonès G., Beltrán R., Caballeria J., Pedro-Botet J., Morcillo-Suárez C., Navarro A., Joven J., Camps J. (2009). The measurement of the lactonase activity of paraoxonase-1 in the clinical evaluation of patients with chronic liver impairment. Clin. Biochem..

[28] Ferré N., Marsillach J., Camps J., Mackness B., Mackness M., Riu F., Coll B., Tous M., Joven J. (2006). Paraoxonase-1 is associated with oxidative stress, fibrosis and FAS expression in chronic liver diseases. J. Hepatol..

[29] Pasqualini L., Cortese C., Marchesi S., Siepi D., Pirro M., Vaudo G., Liberatoscioli L., Gnasso A., Schillaci G., Mannarino E. (2005). Paraoxonase-1 activity modulates endothelial function in patients with peripheral arterial disease. Atherosclerosis.

[30] Rull A., García R., Fernández-Sender L., Beltrán-Debón R., Aragonès G., Alegret J.M., Alonso-Villaverde C., Mackness B., Mackness M., Camps J. (2011). The role of combined assessment of defense against oxidative stress and inflammation in the evaluation of peripheral arterial disease. Curr. Mol. Med..

[31] Rull A., García R., Fernández-Sender L., García-Heredia A., Aragonès G., Beltrán-Debón R., Marsillach J., Alegret J.M., Martín-Paredero V., Mackness B. (2012). Serum paraoxonase-3 concentration is associated with insulin sensitivity in peripheral artery disease and with inflammation in coronary artery disease. Atherosclerosis.

[32] Wang Y., Wang G.Z., Rabinovitch P.S., Tabas I. (2014). Macrophage mitochondrial oxidative stress promotes atherosclerosis and nuclear factor-κB-mediated inflammation in macrophages. Circ. Res..

[33] Reddy S.T., Devarajan A., Bourquard N., Shih D., Fogelman A.M. (2008). Is it just paraoxonase 1 or are other members of the paraoxonase gene family implicated in atherosclerosis?. Curr. Opin. Lipidol..

[34] Mastorikou M., Mackness B., Liu Y., Mackness M. (2008). Glycation of paraoxonase-1 inhibits its activity and impairs the ability of high-density lipoprotein to metabolise membrane lipid hydroperoxides. Diabet. Med..

[35] Mackness B., Beltran-Debon R., Aragones G., Joven J., Camps J., Mackness M. (2010). Human tissue distribution of paraoxonases 1 and 2 mRNA. IUBMB Life.

[36] Draganov D.I., Stetson P.L., Watson D.E., Billecke S.S., la Du B.N. (2000). Rabbit serum paraoxonase 3 (PON3) is a high density lipoprotein associated lactonase and protects low density lipoprotein against oxidation. J. Biol. Chem..

[37] Teiber J.F., Draganov D.I., la Du B.N. (2003). Lactonase and lactonising activities of human serum paraoxonase (PON1) and rabbit serum PON3. Biochem. Pharmacol..

[38] Liu Y., Mackness B., Mackness M. (2008). Comparison of the ability of paraoxonases 1 and 3 to attenuate the *in vitro* oxidation of low-density lipoprotein and reduce macrophage oxidative stress. Free Radic. Biol. Med..

[39] Shih D.M., Xia Y.R., Wang X.P., Bourquard N., Fogelman A.M., Lusis A.J., Reddy S.T. (2007). Decreased obesity and atherosclerosis in human paraoxonase 3 transgenic mice. Circ. Res..

[40] Marsillach J., Camps J., Beltran-Debón R., Rull A., Aragones G., Maestre-Martínez C., Sabench F., Hernández M., Castillo D.D., Joven J. (2011). Immunohistochemical analysis of paraoxonases-1 and 3 in human atheromatous plaques. Eur. J. Clin. Investig..

[41] Satiroglu O., Uydu H.A., Demir A., Bostan M., Atak M., Bozkurt E. (2011). Association between plasma monocyte chemoattractant protein-1 levels and the extent of atherosclerotic peripheral artery disease. Tohoku J. Exp. Med..

[42] Coll B., Alonso-Villaverde C., Joven J. (2007). Monocyte chemoattractant protein-1 and atherosclerosis: Is there room for an additional biomarker?. Clin. Chim. Acta.

[43] Schnabel R.B., Baumert J., Barbalic M., Dupuis J., Ellinor P.T., Durda P., Dehghan A., Bis J.C., Illig T., Morrison A.C. (2010). Duffy antigen receptor for chemokines (Darc) polymorphism regulates circulating concentrations of monocyte chemoattractant protein-1 and other inflammatory mediators. Blood.

[44] Aragones G., Ercilla A., Barreda M., Rull A., Beltrán-Debón R., Rodríguez-Gallego E., Alonso-Villaverde C., Camps J., Joven J. (2012). Human Duffy blood group alloantigen system influences the measurement of monocyte chemoattractant protein-1 (MCP-1) in serum but not in plasma. Clin. Lab..

[45] Reckless J., Rubin E.M., Verstuyft J.B., Metcalfe J.C., Grainger D.J. (1999). Monocyte chemoattractant protein-1 but not tumor necrosis factor-α is correlated with monocyte infiltration in mouse lipid lesions. Circulation.

[46] Graham G.J., Locati M., Mantovani A., Rot A., Thelen M. (2012). The biochemistry and biology of the atypical chemokine receptors. Immunol. Lett..

[47] Manea A., Manea S.A., Todirita A., Albulescu I.C., Raicu M., Sasson S., Simionescu M. (2015). High-glucose-increased expression and activation of NADPH oxidase in human vascular smooth muscle cells is mediated by 4-hydroxynonenal-activated PPARα and PPARβ/δ. Cell Tissue Res..

[48] Fuhrman B. (2012). Regulation of hepatic paraoxonase-1 expression. J. Lipids.

[49] Holvoet P., Rull A., García-Heredia A., López-Sanromà S., Geeraert B., Joven J., Camps J. (2015). Stevia-derived compounds attenuate the toxic effects of ectopic lipid accumulation in the liver of obese mice: A transcriptomic and metabolomic study. Food Chem. Toxicol..

[50] Marsillach J., Camps J., Ferré N., Beltrán R., Rull A., Mackness B., Mackness M., Joven J. (2009). Paraoxonase-1 is related to inflammation, fibrosis and PPARδ in experimental liver disease. BMC Gastroenterol..

[51] Fontaine R., Kim M., Kieny R. (1954). Surgical treatment of peripheral circulation disorders. Helv. Chir. Acta.

[52] Ng C.J., Wadleigh D.J., Gangopadhyay A., Hama S., Grijalva V.R., Navab M., Fogelman A.M., Reddy S.T. (2001). Paraoxonase-2 is a ubiquitously expressed protein with antioxidant properties and is capable of preventing cell-mediated oxidative modification of low density lipoprotein. J. Biol. Chem..

[53] Reddy S.T., Wadleigh D.J., Grijalva V., Ng C., Hama S., Gangopadhyay A., Shih D.M., Lusis A.J., Navab M., Fogelman A.M. (2001). Human paraoxonase-3 is an HDL-associated enzyme with biological activity similar to paraoxonase-1 protein but is not regulated by oxidised lipids. Arterioscler. Thromb. Vasc. Biol..

[54] Marsillach J., Mackness B., Mackness M., Riu F., Beltran R., Joven J., Camps J. (2008). Immunohistochemical analysis of paraoxonase-1, 2 and 3 expression in normal mouse tissues. Free Radic. Biol. Med..

[55] AnalySIS^®^ 3.1. Step by Step Analysis. ftp://ftp.ccmr.cornell.edu/utility/FEI%20temp/AnalySIS%20docs/Getting%20Started.pdf.

[56] Suzme R., Yalcin O., Gurdol F., Gungor F., Bilir A. (2007). Connective tissue alterations in women with pelvic organ prolapse and urinary incontinence. Acta Obstet. Gynecol. Scand..

[57] Palmowski M., Huppert J., Ladewig G., Hauff P., Reinhardt M., Mueller M.M., Woenne E.C., Jenne J.W., Maurer M., Kauffmann G.W. (2008). Molecular profiling of angiogenesis with targeted ultrasound imaging: Early assessment of antiangiogenic therapy effects. Mol. Cancer Ther..

[58] Białas M., Okon K., Czopek J. (2003). Assessing microvessel density in gastric carcinoma: A comparison of three markers. Pol. J. Pathol..

[59] Sander C.S., Hamm F., Elsner P., Thiele J.J. (2003). Oxidative stress in malignant melanoma and non-melanoma skin cancer. Br. J. Dermatol..

