# Supplementary material for: Immunohistochemical Analysis of Paraoxonases and Chemokines in Arteries of Patients with Peripheral Artery Disease
Source: Int J Mol Sci. 2015 May 18;16(5):11323–38. doi: 10.3390/ijms160511323 (PMC4463702; doi:10.3390/ijms160511323)
Supplement: Supplementary file 1 [file ijms-16-11323-s001.pdf]

Supplementary Information

Table S1. Differences in selected variables between control individuals and PAD patients, excluding smokers from the PAD group.

**Parameter**	**Control (*n* = 8)**	**PAD (*n* = 50)**	***p*-Value**
IMT (mm)	1.00 (0.70–1.298)	1.35 (0.99–1.83)	0.133
I/M ratio	0.16 (0.13–0.65)	2.14 (1.42–3.22)	**<0.001**
% PON1 staining	1.70 (1.54–3.72)	11.17 (6.16–15.37)	**<0.001**
% PON3 staining	0.55 (0.22–0.73)	3.34 (2.05–4.55)	**<0.001**
% CCL2 staining	2.26 (0.36–3.65)	30.75 (9.63–43.86)	**<0.001**
% CCR2 staining	18.29 (7.02–27.56)	20.42 (12.79–30.50)	0.650
% CD68 staining	1.10 (0.65–2.88)	4.84 (1.43–9.23)	**0.027**
% D6 staining	0.83 (0.22–12.9)	41.91 (27.85–56.15)	**<0.001**
% DARC staining	3.29 (2.01–5.06)	30.62 (17.79–46.76)	**<0.001**

IMT: Intima-Media thickness. Results are shown as medians (IQR). Staining for CCL2, CCR2, CD68, DARC, D6, PON1 and PON3 were measured as the area of positive staining and expressed as percentage of the total area examined using the image analysis system (see text for details). The bold numbers highlight the statistically significant differences.

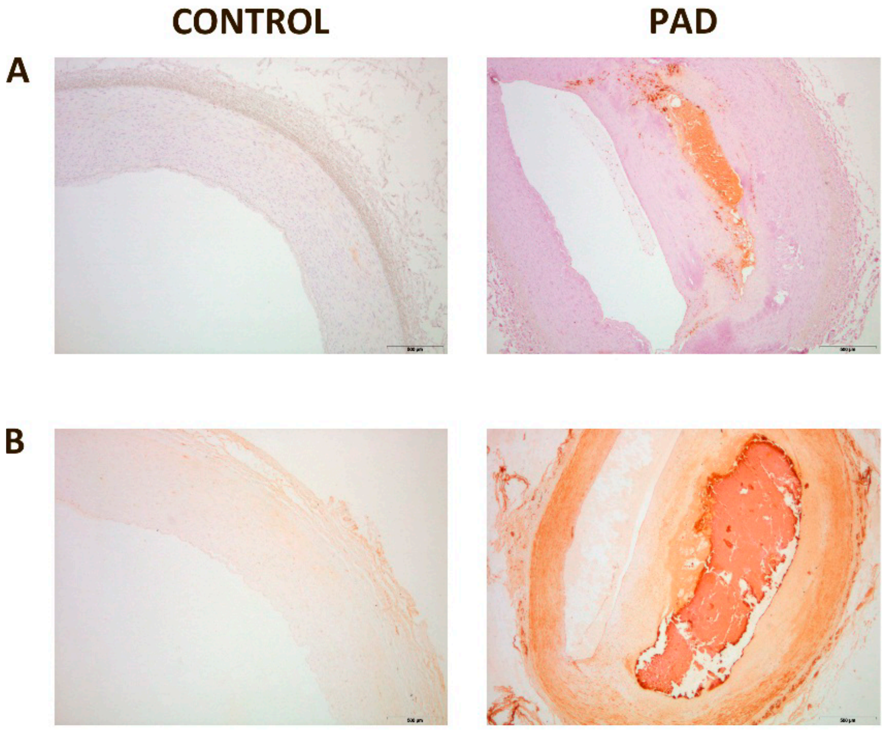

Figure S1. Representative immunohistochemical images of serial sections for CD68 and PON1 staining of peripheral arteries: (**A**) CD68; (**B**) PON1. Both stainings are observed at the lesion site. Magnification 20×.

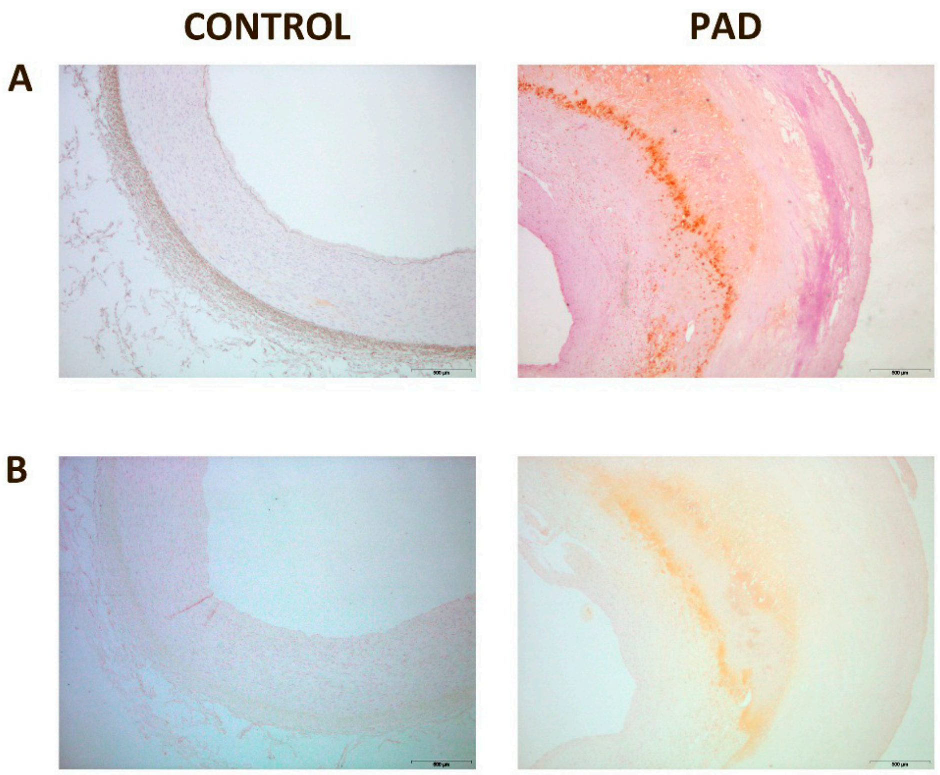

Figure S2. Representative immunohistochemical images of sections from the same tissue block for CD68 and CCL2 staining of peripheral arteries: (**A**) CD68; (**B**) CCL2. Magnification 20×.

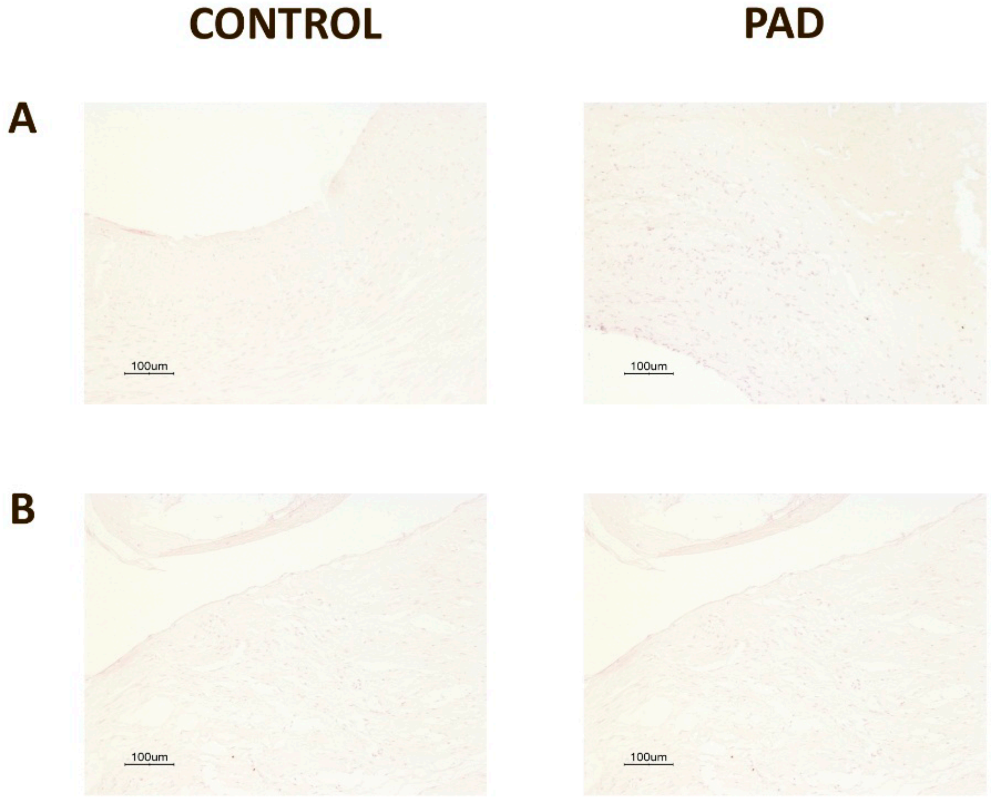

Figure S3. Representative immunohistochemical images of negative controls in control arteries and arteries from patients with peripheral artery disease: (**A**) PON1; (**B**) PON3.

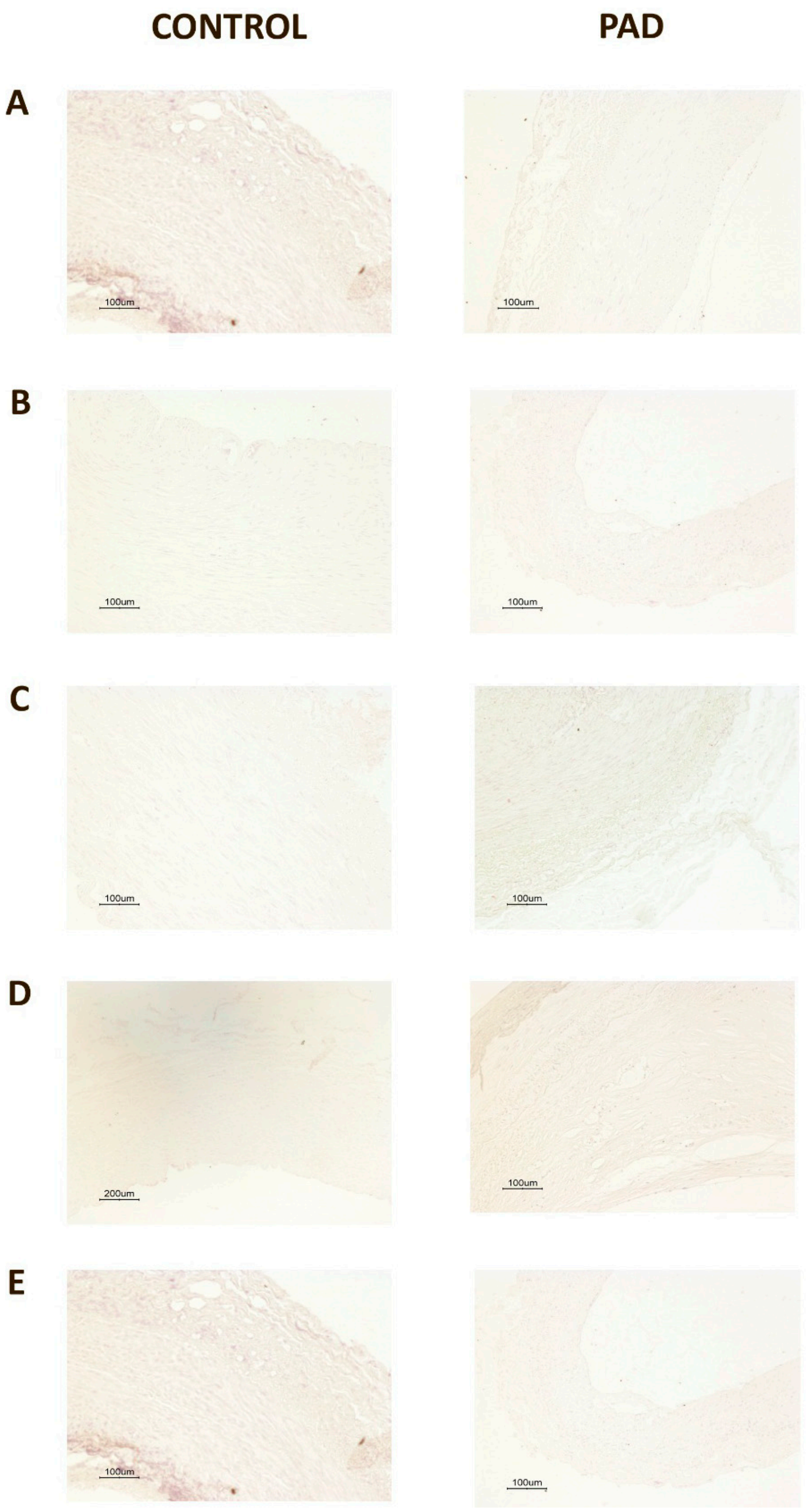

Figure S4. Representative immunohistochemical images of negative controls in control arteries and arteries from patients with peripheral artery disease: (**A**) CCL2; (**B**) CCR2; (**C**) CD68; (**D**) D6; (**E**) DARC.

Supplementary Methods: Phase Analysis with the AnalySIS® System

Objects in true-color images to be displayed in false color are defined with the Set color threshold command. Low and up threshold for the three basic color parameters (red, green and blue) are set and assigned a phase. Thresholds can also be interactively set in the image. To do this, a set of pixels is selected. For each phase, a false color for the respective object is selected. Areas of color ranges not assigned to a phase will not be calculated. This command generates an 8-bit false-color image in the destination image buffer. All gray-value areas assigned to a phase will be colored according to that phase. A measurement sheet is generated containing the absolute areas of the gray-value phases, as well as the percentage area of each phase relative to either the total image area or the area within the active frame. Sheet column headers contain phase name and lower and upper thresholds. The column header's color corresponds to its respective phase. Phase analysis of various gray-value ranges enables to determine the percentage surface area of a particular material on a background. Surface area can be calculated in true-color images using selected color ranges. Summarized from the AnalySIS® User's Guide, freely available at: ftp://ftp.ccmr.cornell.edu/utility/FEI%20temp/AnalySIS%20docs/Getting%20Started.pdf).
